# The Effect of Alpha tACS on the Temporal Resolution of Visual Perception

**DOI:** 10.3389/fpsyg.2020.01765

**Published:** 2020-07-31

**Authors:** Luca Battaglini, Federica Mena, Andrea Ghiani, Clara Casco, David Melcher, Luca Ronconi

**Affiliations:** ^1^Department of General Psychology, University of Padua, Padua, Italy; ^2^Neuro.Vis. U.S. Laboratory, University of Padua, Padua, Italy; ^3^Center for Mind/Brain Sciences, Department of Psychology and Cognitive Science, University of Trento, Rovereto, Italy; ^4^School of Psychology, Vita-Salute San Raffaele University, Milan, Italy; ^5^Division of Neuroscience, IRCCS San Raffaele Scientific Institute, Milan, Italy

**Keywords:** perception, attention, neural oscillations, EEG alpha activity, transcranial AC stimulation

## Abstract

We experience the world around us as a smooth and continuous flow. However, there is growing evidence that the stream of sensory inputs is not elaborated in an analog way but is instead organized in discrete or quasi-discrete temporal processing windows. These discrete windows are suggested to depend on rhythmic neural activity in the alpha (and theta) frequency bands, which in turn reflect changes in neural activity within, and coupling between, cortical areas. In the present study, we investigated a possible causal link between oscillatory brain activity in the alpha range (8–12 Hz) and the temporal resolution of visual perception, which determines whether sequential stimuli are perceived as distinct entities or combined into a single representation. To this aim, we employed a two-flash fusion task while participants received focal transcranial alternating current stimulation (tACS) in extra-striate visual regions including V5/MT of the right hemisphere. Our findings show that 10-Hz tACS, as opposed to a placebo (sham tACS), reduces the temporal resolution of perception, inducing participants to integrate the two stimuli into a unique percept more often. This pattern was observed only in the contralateral visual hemifield, providing further support for a specific effect of alpha tACS. The present findings corroborate the idea of a causal link between temporal windows of integration/segregation and oscillatory alpha activity in V5/MT and extra-striate visual regions. They also stimulate future research on possible ways to shape the temporal resolution of human vision in an individualized manner.

## Introduction

Reducing the complexity of our sensory environment is one of the primary aims of human perception. Considering the temporal domain, although we experience a smooth and continuous perception, the continuous flow of sensory inputs is not thought to be elaborated in a purely analog way, but it is instead segregated or integrated over time (Stroud, 1967). The two processes balance each other in order to achieve an optimal interpretation of sensory input (Dixon and Dilollo, 1994). Specifically, temporal segregation processes are needed to increase perceptual temporal resolution and obtain a higher sensitivity to changes happening in a dynamic environment or determined by our own actions (e.g., eye movements). Temporal integration processes, on the other hand, are necessary to gain the stability of perceptual representations that is fundamental for accurate and precise analysis of objects.

In terms of neurophysiological mechanism, reaching an optimal balance between temporal segregation and integration mechanisms may involve rhythmic oscillatory processes (VanRullen, 2016; White, 2018). This idea was raised in seminal neurophysiological studies showing that perception depends on the rhythmic sampling of sensory inputs (Bishop, 1932; Lansing, 1957; Harter, 1967). More recently, further support to this view comes from non-human primate studies showing that sensory areas fire more frequently in specific neural phases of the local field potential oscillations (Haegens et al., 2011). Human studies that employed electroencephalography (EEG) are in line with this evidence, showing significant relationships between oscillations in the theta (4–7 Hz) and alpha (8–12 Hz) rhythms and sensitivity to incoming sensory input (Busch et al., 2009; Mathewson et al., 2009; Dugue et al., 2011; Fiebelkorn et al., 2013).

Using different types of experimental paradigms to measure the temporal resolution of visual perception, i.e., the ability to perceive sequential stimuli as distinct entities or to combine them into a unique perceptual representation, several human M/EEG studies have provided evidence for a significant relationship between the temporal resolution of perception and rhythmic neural processes in the alpha (8–12 Hz) and theta (4–7 Hz) band (Varela et al., 1981; Mathewson et al., 2012; Wutz et al., 2014, 2016, 2018; Samaha and Postle, 2015; Milton and Pleydell-Pearce, 2016; Ronconi and Melcher, 2017; Ronconi et al., 2017, 2018). Different aspects of ongoing neural oscillations have been linked to temporal mechanisms in visual perception. Some studies show that the phase of ongoing prestimulus oscillations in the alpha band predicts whether successive stimuli are integrated or segregated in time (Varela et al., 1981; Mathewson et al., 2012; Wutz et al., 2014; Milton and Pleydell-Pearce, 2016; Ronconi et al., 2017). In addition, other studies show that faster alpha oscillations in the prestimulus period and in a resting-state condition are associated to better temporal segregation in the unisensory visual modality (Samaha and Postle, 2015; Wutz et al., 2018) and also in multisensory audio-visual tasks (Cecere et al., 2015; Keil and Senkowski, 2017). In parallel, participants showed a slower alpha frequency in trials requiring temporal integration (Wutz et al., 2018). Overall, these results are in line with the idea that temporal integration/segregation mechanism, like any other computation on sensory input that require top-down and feedback loops, is associated to discrete or quasi-discrete temporal processing windows, which in turn depend on rhythmic neural activity in certain frequency bands. The basic idea is that two stimuli falling into the same oscillatory cycle, or into the same favorable phase of a cycle, are bound into a unique percept. Contrarily, two stimuli falling into separate cycles are parsed into two different temporal events (Wutz and Melcher, 2014; VanRullen, 2016; White, 2018).

The accumulation of correlational evidence leads to the question of whether a causal relationship can be demonstrated between oscillatory brain activity and temporal mechanisms of perception. Following the observation that sensory stimulation (visual and/or auditory) at specific frequencies can entrain ongoing neural oscillations causing a modulation of perceptual phenomena (Thut et al., 2011; Mathewson et al., 2012; de Graaf et al., 2013; Spaak et al., 2014; for a review, see Herrmann et al., 2016), recent studies have provided the first observations that temporal segregation/integration can be influenced by the alignment of the ongoing oscillatory activity to a particular rhythm even when the entrainment period is only a few seconds (Ronconi and Melcher, 2017; Ronconi et al., 2018; Chota and VanRullen, 2019). The importance of the evidence showing that sensory input can entrain brain oscillations and modulate perception is to demonstrate that a short-lasting plasticity characterizes the perceptual mechanism of temporal integration and segregation, which in turn holds promise for more long-lasting entrainment protocols.

Transcranial alternating current stimulation (tACS) is another approach that has the potentiality to shed light on the causal link between neural oscillations and perception. Some studies demonstrate that tACS, possibly driving the activity of cortical regions to the external frequency imposed by tACS, represents an efficient method to determine a significant entrainment of the brain’s cortical oscillations during and after the stimulation (Fröhlich and McCormick, 2010; Helfrich et al., 2014b). There is, however, an alternative explanation for the effects of tACS that last beyond stimulation (i.e., offline to tACS). The mechanism of action behind tACS after-effects would involve synaptic changes via spike-timing-dependent plasticity^1^ rather than entrainment (Vossen et al., 2015; Vosskuhl et al., 2018).

While further neurophysiological investigation is needed to clarify the precise nature of the (online and offline) interaction between tACS and cortical rhythms, increasing evidence demonstrates the possibility of modulating perceptual and cognitive processes with tACS (Herrmann et al., 2016). For instance, stimulating the right parietal cortex with tACS at theta frequency can increase the capacity of visual working memory (Wolinski et al., 2018; Bender et al., 2019), stimulating the parieto-occipital cortex with tACS in the gamma frequency can modulate bistable motion perception (Helfrich et al., 2014a; Strüber et al., 2014), and stimulating the parietal cortex with a sinusoidal current within the beta band can ameliorate perception of objects within a crowding regime (Battaglini et al., 2020).

To date, very few studies have used tACS to show a causal link between alpha rhythms in specific brain areas and the temporal resolution of perception. In the context of visual perception, Minami and Amano (2017) used a motion-induced spatial conflict task where subjects reported illusory visual vibrations that could be modulated by tACS: i.e., the perceived jitter frequency changed with the frequency of tACS within the alpha band. Extending the analysis of the previous literature to the context of multisensory perception, Cecere et al. (2015) evaluated the effects of posterior tACS in the sound-induced double flash illusion, reporting that a slower alpha tACS enlarged the temporal window of the illusion whereas a faster alpha tACS had opposite outcomes.

In the present study, we wanted to bring additional causal evidence about the relationship between oscillatory alpha activity and the temporal resolution of perception. At the same time, we wanted to provide a more precise identification of cortical areas that are relevant for timing in perception and that are specifically involved in this computationally relevant rhythm. Indeed, while on the one hand increasing M/EEG studies found associations between alpha and timing in visual perception, as extensively reviewed above, the identification of a cortical “perceptual alpha network” remains rather elusive. If alpha is a relevant cortical rhythm for temporal perception, we should expect that perceptual timing would not be affected by rhythmic stimulation far beyond the alpha band (e.g., in the beta band). Accordingly, the main hypothesis of the present study was that 10-Hz tACS as compared to sham and 18-Hz tACS could influence the temporal resolution of perception. To test this, we employed a two-flash fusion task, in which the perception of two successive stimuli has been previously shown to correlate with the individual alpha frequency (IAF) as measured with EEG in the prestimulus time period (Samaha and Postle, 2015; see also Wutz et al., 2018 for similar findings) or during a resting state (Samaha and Postle, 2015) and with the phase of ongoing EEG alpha oscillations (Ronconi et al., 2017). The two-flash fusion task has also been shown to be significantly modulated by pre-stimulus sensory entrainment in the alpha band (Ronconi and Melcher, 2017). We used focal multi-channels tACS at 10, at 18 Hz, or within a sham (placebo) regime to stimulate extra-striate visual regions centered on V5/MT, of the right hemisphere. The choice of these areas is driven by the topographical distribution of EEG evidence showing that alpha frequency in posterior regions is correlated to temporal integration/segregation in the two-flash fusion task (Samaha and Postle, 2015; Ronconi et al., 2017). It is also in line with theoretical models proposing the existence of a “when” pathway in the human visual system involving V5/MT (Battelli et al., 2007) and, more generally, to experimental evidence linking V5/MT to timing processes in the visual domain (Bueti et al., 2008a, b; Salvioni et al., 2013).

## Materials and Methods

### Participants

Thirty adult participants (mean age = 23.5, SD = 2.71, 14 males) recruited at the University of Padua took part in the present study providing their informed consent. All observers had normal or corrected-to-normal vision and were all naïve with respect to the purpose of the experiment. None of the participants reported history of neurological and/or psychiatric disorders. All the participants fell within the criteria for the administration of tACS (Antal et al., 2017). They were asked at the end of the experiment whether they could guess the presence of stimulation (see questionnaire in Fertonani et al., 2015). The guessing rate was at chance level (see Supplementary Material). This study was approved by the Ethics Committee of the Department of General Psychology of the University of Padua (protocol 3065).

### Stimuli and Apparatus

All visual stimuli were displayed binocularly on a 22-in CRT screen at 1024 × 768-pixel spatial resolution and a vertical refresh rate of 100 Hz, at a viewing distance of 57 cm. The stimuli were displayed on a mid-level gray screen with ∼50 cd/m^2^ luminance. A gamma correction was applied so that luminance was a linear function of the digital representation of the image.

All visual stimuli were created with Psychtoolbox for Matlab (Brainard, 1997; Pelli, 1997; Kleiner et al., 2007). The target stimuli (hereafter also referred to as “flashes”) were luminance-defined Gaussian blobs sized 0.5° × 0.5°. We first determined with a QUEST procedure the absolute contrast threshold; the resulting contrast value was then multiplied by a factor of 1.5. The choice to use relatively weak stimuli, but still supra-threshold, derives from the hypothesis that this particular setting is thought to increase the sensitivity to the effects of the ongoing oscillations in the alpha frequency range as shown in previous studies (Ronconi and Melcher, 2017; Ronconi et al., 2017). Indeed, a full contrast stimulus might create a phase reset of ongoing neural oscillation and thus decrease the influence of tACS. At the same time, using stimuli well above the absolute contrast threshold is important in order to ensure that the stimulus was highly visible and so the response “one flash” was determined just by the occurrence of an integration process.

### Procedure

We implemented a within-subject design in which every participant took part in three tACS sessions taking place in three different days. On each session, participants performed the two-flash fusion task (see the details below) while receiving tACS at a frequency within the alpha band (10 Hz), within the beta band (18 Hz), or within a sham (control) regime. The order of the three sessions was counterbalanced across participants. Each session lasted ∼51 min, in addition to the time spent for the montage of the tACS/EEG apparatus, and was structured as follows: initially, 3 min of pre-stimulation eye-closed resting-state EEG was recorded; then, each participant performed 45 min of the two-flash fusion task while receiving tACS stimulation; finally, 3 min of post-stimulation eye-closed resting-state EEG was recorded. The estimation of the absolute contrast threshold for each participant was performed only at the beginning of the first session (first day).

**FIGURE 1:**
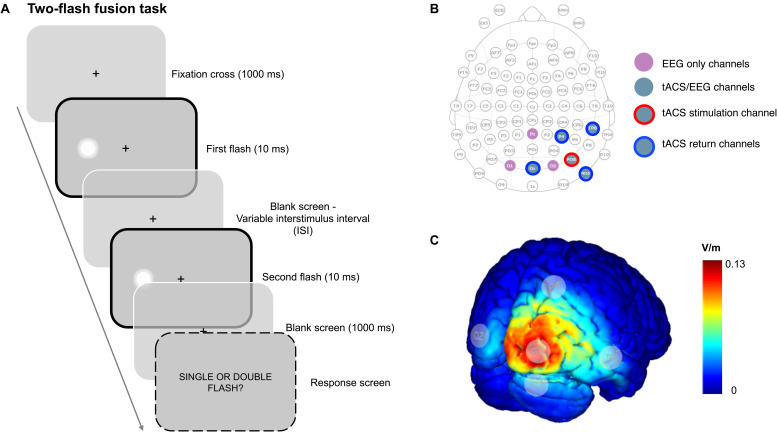
**(A)** Illustration of a trial example and of the two-flash fusion task employed in the present study. **(B)** tACS/EEG montage. **(C)** tACS voltage distribution on the cortical surface; as it can be seen, the maximum voltage was delivered on the right extra-striate visual areas including V5/MT, in agreement with previous studies stimulating in PO8 (stimulation electrode was placed in PO8) (Zito et al., 2015).

Regarding the two-flash fusion task (Figure 1A), all trials started with a central fixation point displayed for 1000 ms, after which the two target stimuli were randomly presented in the left or right hemifield at 7° of eccentricity from the fixation point, aligned to the horizontal axis (the hemifield of appearance of the targets was randomized across trials). The two target flashes, each displayed for 10 ms, were separated by seven different inter-stimulus interval (ISI) between the first and the second flash (20, 30, 40, 50, 60, 70, and 80 ms). According to previous studies, an ISI around 44 ms should correspond to the threshold at which perception changes between one flash (integration) and two flashes (segregation) (Ronconi and Melcher, 2017; Ronconi et al., 2017). After 1500 ms from the target presentation, the response screen appeared and participants were asked to indicate whether they perceived one or two flashes by pressing the corresponding button on the keyboard using both the right and left hand and with no temporal constraints. The following trial started after an intertrial interval of 1000 ms.

The fixation of participants was not controlled with the eye-tracker but, given that stimuli could appear randomly in the left or right hemifield, participants could not anticipate the location of the stimuli. Moreover, different studies demonstrated that ∼100 ms of presentation is very unlikely to be sufficient to perform saccadic movements toward the stimulus (Carpenter, 1988; Martinez-Conde et al., 2004; Wright and Ward, 2008). The total amount of trials administered on average for each participant was 713 ± 93 (mean ± SD) across tACS sessions, 102 ± 14 for each ISI.

### tACS Stimulation Setting

Transcranial alternating current stimulation was delivered through a StarStim8 device from Neuroelectrics^2^. StarStim8 is a hybrid tACS/EEG system with eight channels that can be located in 39 possible scalp positions according to the 10-10 system. For the present study, we employed five PISTIM Ag/AgCl electrodes with 1 cm radius both for delivering focal tACS and EEG recording. Moreover, three GELTRODE Ag/AgCl electrodes were used just for EEG recording. As displayed in Figures 1B,C, the stimulation electrode was placed in PO8, while the four return electrodes were placed in Oz, P4, PO10, and TP8. This montage was chosen after carefully evaluating the electric field distribution with the software NIC 2.0; in particular, according to previous EEG studies that underlined right posterior areas as the main cortical sources involved in the two-flash fusion task (Samaha and Postle, 2015; Ronconi et al., 2017) and, importantly, based on previous evidence using transcranial direct current stimulation (tDCS) that reported a significant modulation of motion perception using these same electrodes, positioning centered on PO8 (Zito et al., 2015). Three protocols with different stimulation frequencies were created, 10 Hz, 18 Hz, and Sham. Stimulation intensity was set at 0.8 mA (milliampere), with offset set at 0 mA (distance peak-to-peak of 1.6 mA); this value was chosen following the most recent guidelines for tACS safety guidelines (Antal et al., 2017). At the beginning of the tACS session, electrical current intensity was linearly ramped up from 0 to ±0.8 mA over 20 s. The full ±0.8 mA current was then applied for 45 min, after which the current amplitude was ramped back down from ±0.8 mA to zero over 20 s. In the sham session, the current ramped up from 0 to ±0.8 mA at a frequency of 10 Hz over 20 s and remained at full power only for 10 s before ramping back down to zero. Current amplitude ramped up and down in the same way at the end of the session.

### Resting-State EEG Recording and Pre-processing

Three minutes of eye-closed resting-state EEG was recorded before and after tACS at a sampling frequency of 500 Hz and with a 24-bit digitization using eight electrodes positioned on the following scalp locations: Pz, O1, Oz, O2, P4, PO8, PO10, and TP8. Channels activity was online referenced to Cz and the impedance was kept below 10 kΩ. We did not apply a re-reference because of the low number of channels and their non-uniform spatial distribution. During the resting-state recording, the subjects were asked to close their eyes and relax in the dimly dark room in which any kind of external noise was prevented.

Data analysis was performed offline using Matlab (MathWorks, Inc., Natick, MA, United States) and EEGLAB (Delorme and Makeig, 2004). Specifically, the continuous EEG data were filtered between 0.05 and 40 Hz (Butterworth filter, order = 2) and segmented into 1-s epochs to obtain a total of 180 epochs for both pre- and post-tACS periods. These epochs were visually inspected to remove data segments contaminated by muscular or ocular artifacts (mean ± SD of cleaned epochs: 178.86 ± 3.74 across all conditions). In a few cases, we performed an independent component analysis (ICA) combining pre- and post-tACS data to correct for electrodes artifacts; overall, a single ICA component was removed in seven out of 180 EEG sessions.

From the cleaned epochs, we extracted the FFT spectrum, after applying zero padding (*N* = 2000 samples) to increase the frequency resolution. Data were baseline-normalized (dB) to the average epoch power, before extracting the IAF values for each of the tACS sessions.

Hereafter, we refer to IAF to indicate the frequency corresponding to the peak of the FFT power between 8 and 14 Hz. Differences in resting-state IAF have been shown to correlate with performance in the two-flash fusion task (Samaha and Postle, 2015) and in multimodal tasks measuring the temporal resolution of perception (Cecere et al., 2015; Keil and Senkowski, 2017). Thus, we might expect that individual differences in IAF can determine a different effect of 10-Hz tACS, and thus extracting IAF values before each tACS session is important to account for this potential confounding variable in our analyses.

Due to technical problems, resting-state EEG data were not available for one participant in the Sham tACS condition and one participant in the 18-Hz tACS condition.

### Data Analysis

The proportion of “two flashes” response was analyzed with two different approaches.

In the first approach, we used it as the dependent variable in separate repeated-measures analyses of covariance (ANCOVA) in order to test the hypothesis that tACS within the alpha band, but not beta band, could influence the performance in the two-flash fusion task. The main analysis had three within-subject factors: Hemifield (Left vs. Right), Stimulation session (10 Hz vs. Sham), and ISI (seven levels). We included as a covariate the IAF values, which previous studies showed to be linked in integration/segregation perception processes (Samaha and Postle, 2015; Ronconi et al., 2018), measured from the channel PO8 (the same channel used for the stimulation) as measured before the application of 10-Hz tACS. In particular, the advantage of including the raw IAF value as opposed to the absolute difference between the IAF and the target tACS frequency (which would follow Arnold’s tongue principle; see, for example, Fröhlich, 2015) is to maintain information about a possible asymmetry in the effect. Indeed, it might be possible that tACS is more effective when an individual’s endogenous frequency is above rather than below the target frequency, or vice versa.

A control analysis was performed using a similar ANCOVA, with the within-subject factors Hemifield (Left vs. Right), Stimulation session (18 Hz vs. Sham), and ISI (seven levels); we used as a covariate the IAF values measured from the channel PO8 before the application of 18 Hz tACS. Finally, we compared directly 10-Hz tACS and 18-Hz tACS with two ANCOVAs, with the within-subject factors Hemifield (Left vs. Right), Stimulation session (10 Hz vs. 18 Hz), and ISI (seven levels); in one ANCOVA, we used as a covariate the IAF measured from the channel PO8 before the application of 10-Hz tACS, and in the other ANCOVA, we used as a covariate the IAF measured from the channel PO8 before the application of 18 Hz tACS.

In the second approach, for each participant, and separately for each tACS session and hemifield, we fitted the proportion of two flash responses as a function of ISI with a psychometric curve (logistic), where the lower bound was set at 0 and the higher bound was set at 1 (*y* = 0 means that two flashes were never perceived, and *y* = 1 means that they were always perceived). The only free parameters of the function were *b* (the function slope) and *t* (the 50% threshold), which were restricted to assume values above 0. The formula used was the following:

$$y=\frac{1}{1+e^{b\cdot \left(t-x\right)}}$$

In this equation, *x* represents the ISI between the two flashes and *y* represents the proportion of “two flashes” responses. The individual performance was recomputed to 75% threshold in line with previous studies using two-flash fusion (Samaha and Postle, 2015) or other temporal segregation tasks (Ronconi et al., 2020). The psychometric approach was used to test whether segregation performance increased differentially as a function of ISI in the different tACS sessions. Regarding behavioral data, four participants were excluded from the analyses for the following reasons: two participants were excluded because they reported a proportion of “two flashes” responses in the sham condition, which was on average (across all ISIs) below 2 SD relative to the observed mean of the sample, thus constituting two outliers; two other subjects were excluded because their individual psychometric fitting procedure resulted to be worse as compared to the fit obtained with a simple horizontal line in the majority of the tACS sessions, indexing that the proportion of “two flashes” response did not increase as a function of ISI as expected. Thus, the final sample in which we conducted the statistical analyses of the effect of tACS on behavioral performance was composed of 26 participants (mean age = 23.3, SD = 2.31, 11 males).

Finally, we tested whether the resting-state EEG power in the alpha band (8–12 Hz) changed after 10-Hz tACS as compared to the sham stimulation. To this aim, we run an ANCOVA using as within-subject factor the Stimulation session (two levels: 10 Hz vs. Sham), the Time of recording (pre- vs. post-tACS), and the Channels (eight levels corresponding to the eight channels used for EEG recording). We included as a covariate the IAF values measured from the channel PO8 (the same channel used for the stimulation).

## Results

In our sample, the average IAF calculated from the stimulated area (channel PO8) during the 10-Hz tACS condition was 10.2 Hz (SD = 0.9) and the majority of participants (65.4%) showed an IAF value between 9 and 11 Hz.

**FIGURE 2:**
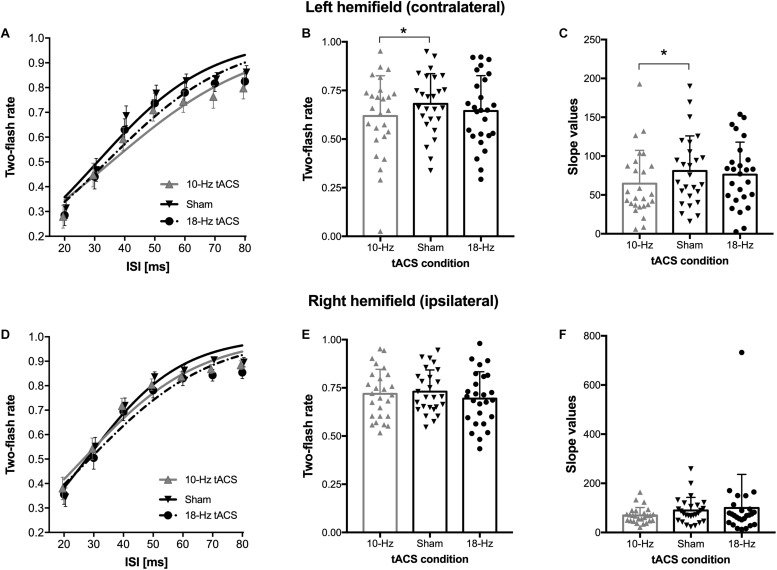
Effect of tACS on temporal integration/segregation performance. Average two-flash response rate as a function of inter-stimulus interval (ISI) and relative psychometric functions for the **(A)** left/contralateral and the **(D)** right/ipsilateral hemifield trials obtained while participants received 10-Hz, 18-Hz, or sham tACS. Two-flash response rate (averaged across ISIs) as a function of tACS conditions for the **(B)** left/contralateral and the **(E)** right/ipsilateral hemifield trials, with dots representing individual observations and bars showing group average. Slope values of the psychometric curve (logistic) obtained from the individual data as a function of tACS conditions for the **(C)** left/contralateral and the **(F)** right/ipsilateral hemifield trials, with dots representing individual observations and bars showing group average. ^∗^ Indicates *p*-values < 0.05. Error bars represent the SEM.

The ANCOVA testing the specificity of 10-Hz vs. sham tACS, while controlling for the differences in resting-state IAF, revealed a main effect of ISI [*F*_(__6_,_144__)_ = 3.23, *p* = 0.005, η^2^_*p*_ = 0.12], showing that the “two flashes” response rate increased as a function of the temporal interval between the two stimuli as expected. Importantly, we found a significant three-way interaction Hemifield × Stimulation session × IAF [*F*_(__1_,_24__)_ = 4.5, *p* = 0.044, η^2^_*p*_ = 0.16]. No other main effects or interaction became significant. To explore this interaction, we performed planned comparisons (one-tailed *t*-tests), indicating that in the visual hemifield contralateral to the stimulation site (i.e., left hemifield), there was a significant decrease in the proportion of two flashes perceived during the 10-Hz tACS as compared to sham tACS [*t*_(__25__)_ = −2.27, *p* = 0.032; mean ± SEM for 10-Hz and sham tACS were 0.62 ± 0.04 and 0.68 ± 0.03, respectively; Figures 2A,B]. No significant difference emerged in the ipsilateral hemifield [*t*_(__25__)_ = −0.60, *p* = 0.554; Figures 2D,E].

The control ANCOVA testing 18 Hz vs. sham tACS, while controlling for the differences in IAF, revealed again a main effect of ISI [*F*_(__6_,_138__)_ = 6.97, *p* = 0.001, η^2^_*p*_ = 0.15]. Notably, no other main effects or interactions became significant (all *p*s > 0.16). The ANCOVA testing the specificity of 10-Hz vs. 18-Hz tACS, while controlling for the differences in IAF measured before 10-Hz tACS, did not reveal significant interactions, whereas the ANCOVA testing the specificity of 10-Hz vs. 18-Hz tACS, while controlling for the differences in IAF measured before 18-Hz tACS revealed the three-way interaction Hemifield × Stimulation session × IAF [*F*_(__1_,_23__)_ = 8.13, *p* = 0.009, η^2^_*p*_ = 0.26]. This result supports, at least partially, a specificity of 10-Hz vs. 18-Hz tACS. On the left hemifield, segregation rate was lower for 10-Hz tACS than for 18-Hz tACS, while the opposite was true for the ipsilateral hemifield where 10 Hz led to higher two flash rate than 18 Hz; planned comparisons were not significant (*p*s > 0.27).

The analysis of the psychometric function, which considered the slope and 75% threshold value calculated on the individual data (see the details above), provided results that were in agreement with the ANCOVA results reported above. While 75% threshold value did not differ between 10-Hz and sham tACS [one-tailed *t*-test: *t*_(__25__)_ = 1.44, *p* = 0.08], we found a significant difference in the slope [one-tailed *t*-test: *t*_(__25__)_ = −1.98, *p* = 0.029; Figures 2C,F]. Specifically, the task performed with 10-Hz tACS was characterized by a shallower slope of the psychometric curve of the proportion of two-flash responses as a function of the ISI between the two targets (mean ± SEM for 10-Hz and sham tACS were 64.5 ± 8.4 and 80.88 ± 8.84, respectively).

The analysis on resting-state EEG power revealed no main effect of stimulation and, importantly, no interactions involving the factor Stimulation (all *p*s > 0.1), suggesting that although a behavioral effect emerged during the application of 10-Hz tACS, this effect was not accompanied by variations in the alpha rhythms as measured by the resting-state EEG after the end of the stimulation.

## Discussion

Correlational evidence obtained with M/EEG is increasing, suggesting that ongoing neural oscillations in the alpha band are linked to temporal aspects of perception. In particular, alpha oscillations would index whether successive stimuli are integrated into a single percept or rather segregated in separate events (Varela et al., 1981; Mathewson et al., 2012; Wutz et al., 2014, 2018; Samaha and Postle, 2015; Milton and Pleydell-Pearce, 2016; Ronconi and Melcher, 2017; Ronconi et al., 2017). In the present study, we aimed to support a possible causal relation between temporal windows of integration/segregation and the neural mechanisms supporting the temporal resolution of visual perception both in terms of brain areas/network involved and in terms of the precise oscillatory code. To this aim, we took advantage of a recently introduced non-invasive brain stimulation method, i.e., tACS, which has been proposed as a way to shape perceptually relevant brain oscillations with a significant impact on perceptual and cognitive processes (Neuling et al., 2012; Helfrich et al., 2014b; Cecere et al., 2015; Stonkus et al., 2016; Wolinski et al., 2018; Battaglini et al., 2020).

Our results suggested that 10-Hz tACS was able to influence temporal segregation/integration when applied on extra-striate visual areas centered on V5/MT. In particular, participants reported a reduced segregation (or increased integration) ability as determined by the lower proportion of two-flash responses. We also found a significant influence of the individual alpha rhythm (i.e., IAF, which indexes the “speed” of the alpha rhythm) in determining the effect of tACS. Moreover, we stimulated the right occipital–temporal cortex and found evidence that the effect of 10-Hz tACS was specific for stimuli appearing in the contralateral (i.e., left) visual hemifield. This hemifield-specific effect has been demonstrated in other studies using tACS (Wolinski et al., 2018; Bender et al., 2019; Battaglini et al., 2020), and it allows us to exclude the idea that the results could be due to a differential feeling of sham from real stimulation or any other unspecific effect that would have been evident in both visual hemifields. Finally, our active control condition with tACS delivered at a frequency outside the alpha band (i.e., 18 Hz), while seemingly involved in some other aspects of vision and attention, did not significantly modulate temporal integration/segregation performance, in line with previous evidence showing that sensory entrainment at the alpha and theta rhythms, but not beta, is correlated with temporal organization of perception (Ronconi et al., 2016, 2018; Ronconi and Melcher, 2017; Chota and VanRullen, 2019).

These findings, collectively, constitute preliminary evidence about the cortical areas involved in the temporal integration/segregation of simple visual stimuli. We used a focal tACS protocol where electrodes positioning was optimal to stimulate V5/MT, based on previous studies using electrical neurostimulation that found a significant modulation of motion perception with tDCS (Zito et al., 2015). Although we used focal tACS, electrical current was potentially affecting extra-striate visual areas other than V5/MT. Nonetheless, V5/MT seems to be the area that most likely contributes to the behavioral effects on temporal segregation. Indeed, we found this relation not only based on the specific electrodes positioning employed but also considering previous experimental evidence and theoretical proposals that linked V5/MT to timing processes in the visual modality (Battelli et al., 2007; Bueti et al., 2008a, b; Salvioni et al., 2013).

The present findings are in line with many evidence showing that neural oscillations determine periods of increase and decrease in neural excitability that would constitute a primary sampling mechanism of human perception (Jensen et al., 2012, 2015; VanRullen, 2016). Indeed, virtually all computations on sensory input, in particular those involving top-down and feedback loops, lead to discrete or quasi-discrete processing windows, influencing the output of specific sensory computation (e.g., segregation/integration of incoming visual stimuli). It cannot be excluded, however, that variations of neural excitability could be sufficient to account for fluctuations in the temporal resolution of perception without requiring the hypothesis of a temporal window (Milton and Pleydell-Pearce, 2016; Ronconi and Melcher, 2017). Independently of the precise nature of temporal sampling, what the literature increasingly suggests is that such properties are not specific to the visual domain, but they characterize also temporal sampling in the auditory (Poppel, 1997; Pöppel, 2009) and tactile (Fujisaki and Nishida, 2009) modalities, as well as in the multisensory domain (Wallace and Stevenson, 2014; Murray et al., 2016). Our findings show that 10-Hz tACS can influence this fundamental sampling mechanism inducing participants to segregate the two stimuli less frequently (and thus integrate them more often). This evidence corroborates the idea of a causal link between temporal windows of integration/segregation and neural oscillations in the alpha band. Future studies would need to further clarify whether, as suggested by one study at present in the visual domain (Minami and Amano, 2017), tACS tailored on the individual endogenous alpha rhythm can be used to effectively shrink or expand temporal windows in perception. This is particularly important considering also that abnormalities in temporal processing have been shown in a wide range of neurodevelopmental and psychiatric disorders, such as, for example, autism spectrum disorder (Brock et al., 2002; Stevenson et al., 2016), developmental dyslexia (Wallace and Stevenson, 2014; Ronconi et al., 2020), schizophrenia (Wallace and Stevenson, 2014), and attention deficit hyperactivity disorder (Panagiotidi et al., 2017).

It is important to acknowledge also some limitations of the present study. We used a fixed value to stimulate in the alpha range (10 Hz); however, some previous EEG studies linked temporal integration/segregation in perception with the IAF (Samaha and Postle, 2015; Ronconi et al., 2018), so 10 Hz might not be the most appropriate frequency to modulate temporal integration/segregation for all the participants. Nonetheless, the average IAF was very close to the target tACS frequency (see section “Results”) and, moreover, the majority of participants (65.4%) have an IAF value in the range 10 Hz ± 1. Another limitation was the lack of a control montage stimulating a different site, with the same frequencies used in this study, to exclude (subthreshold) sensory entrainment (cutaneous or retinal) and unconscious lateralized effect on spatial attention. Although these alternative accounts cannot be excluded directly from the current experimental design, there are some evidences in contrast with it. First, in the present experiment, none of the participants reported the perception of phosphenes with our tACS Montage. Second, Ronconi and Melcher (2017) used a supra-threshold audio–visual bilateral entrainment and did not find a lateralization effect. Finally, assuming that subthreshold retinal stimulation was caused by our tACS protocol, due to the spreading of the current on the surface of the scalp, the effect would be expected in both hemifields rather than being lateralized as found here^3^.

Moreover, strong interpretation from some of the current results should be made with caution. In particular, it is noted that the stimulation effect was observed only when the covariate was added in the analysis (i.e., tACS effect depends on the IAF values). Also, the statistical difference between 10-Hz tACS and 18-Hz tACS was observed when considering IAF measured before 18-Hz tACS, but not when considering IAF measured before 10-Hz tACS. This only partially suggests a specificity effect of 10-Hz tACS vs. 18-Hz tACS.

In sum, the present study provides initial evidence that focal oscillatory electrical stimulation over extra-striate visual areas including V5/MT at a frequency within the alpha band is capable of shifting the phenomenological timing of visual events. This result will stimulate future investigations on potential ways to influence the temporal resolution of human vision in a controlled manner, taking into account individual differences in the endogenous alpha rhythms that, as suggested also by our results, might be an important mediator of any attempt to modulate conscious perception with external electrical (or magnetic) forces.

## Data Availability Statement

The raw data supporting the conclusions of this article will be made available by the authors, without undue reservation.

## Ethics Statement

The studies involving human participants were reviewed and approved by the Ethics Committee of the Department of General Psychology of the University of Padua (protocol 3065). The patients/participants provided their written informed consent to participate in this study.

## Author Contributions

LB: conceptualization, investigation, methodology, formal analysis, data curation, writing—original draft, visualization, and writing—reviewing and editing. FM and AG: investigation, data curation, and writing—original draft preparation. CC: project administration, resources, supervision, writing—original draft, and writing—reviewing and editing. DM: project administration, methodology, resources, supervision, writing—original draft, and writing—reviewing and editing. LR: conceptualization, methodology, formal analysis, supervision, writing—original draft preparation, visualization, and writing—reviewing and editing. All authors contributed to the article and approved the submitted version.

## Conflict of Interest

The authors declare that the research was conducted in the absence of any commercial or financial relationships that could be construed as a potential conflict of interest.

## Footnotes

1 Spike-timing-dependent plasticity (STDP) is a form of synaptic plasticity in which the temporal order of pre- and post-synaptic spikes determines the direction of plasticity. Long-term potentiation is induced if the pre-synaptic spike in a recurrent network precedes the postsynaptic spike, whereas long-term depression of the postsynaptic spike precedes the presynaptic spike (Buonomano and Carvalho, 2009). This mechanism has been suggested to underlie the enhancement of oscillatory brain activity after tACS (Vossen et al., 2015).

2 http://www.neuroelectrics.com/products/software/nic2/

3 There is a recent literature showing that tACS effects on motor system can be caused by transcutaneous stimulation of peripheral nerves in the skin (Asamoah et al., 2019). However, it was also found that rhythmic activity from peripheral nerves in turn then entrains cortical neurons (Asamoah et al., 2019). To the best of our knowledge, there are no articles showing that tACS effects on the visual system can be caused by nerve stimulation. In addition, Krause et al. (2019) presented data that are difficult to reconcile with Asamoah et al.’s (2019) hypothesis, reporting that tACS can actually entrain single neuron activity in the primate brain even in deep brain structures (Krause et al., 2019).
