# Supplementary material for: The Effect of Alpha tACS on the Temporal Resolution of Visual Perception
Source: Front Psychol. 2020 Jul 31;11:1765. doi: 10.3389/fpsyg.2020.01765 (PMC7412991; doi:10.3389/fpsyg.2020.01765)
Supplement: Supplementary file 1 [file Table_1.docx]

***Supplementary Material***

Participants were asked at the end of the experiment whether they could guess the presence of real vs. sham (placebo) stimulation (1 = real stimulation, 0 = sham/placebo). If participants responded at a chance level, then the observed frequency of “real stimulation” responses” should be not statistically different from 13 in all the stimulation conditions. This was confirmed by a Pearson’s Chi-Square test for frequency distribution (χ^2^_(2)_ = 0.15, p = .99).

***Table 1.***

|  | **18-Hz**  **tACS** | **10-Hz tACS** | **Sham stim.** |
| --- | --- | --- | --- |
| p_1 | 1 | 0 | 0 |
| p_2 | 0 | 0 | 1 |
| p_3 | 0 | 0 | 0 |
| p_4 | 1 | 1 | 1 |
| p_5 | 1 | 0 | 0 |
| p_6 | 1 | 1 | 0 |
| p_7 | 0 | 1 | 0 |
| p_8 | 0 | 1 | 0 |
| p_9 | 1 | 1 | 1 |
| p_10 | 1 | 1 | 0 |
| p_11 | 0 | 0 | 0 |
| p_12 | 0 | 0 | 0 |
| p_13 | 1 | 0 | 1 |
| p_14 | 0 | 1 | 1 |
| p_15 | 0 | 1 | 1 |
| p_16 | 0 | 0 | 0 |
| p_17 | 0 | 1 | 1 |
| p_18 | 0 | 1 | 0 |
| p_19 | 0 | 0 | 1 |
| p_20 | 1 | 0 | 1 |
| p 21 | 1 | 0 | 0 |
| p 22 | 1 | 0 | 1 |
| p 23 | 1 | 1 | 1 |
| p 24 | 1 | 1 | 0 |
| p 25 | 1 | 1 | 1 |
| p 26 | 1 | 0 | 0 |

Moreover, participants completed the questionnaire reported in Fertonani et al., 2015. Tables 2 summarised the results.

Table 2. Percentage of skin sensation

|  |  | 18-Hz  tACS | 10-Hz tACS | Sham stim. |
| --- | --- | --- | --- | --- |
| Itching |  | 23% | 38% | 34% |
| Pain |  | 0% | 0% | 0% |
| Burning |  | 4% | 0% | 4% |
| Warm/Heat |  | 4% | 4% | 4% |
| Pinching |  | 12% | 12% | 0% |
| Metal/Iron taste | | 0% | 0% | 0% |
| Fatigue |  | 4% | 4% | 8% |
| Other |  | 0% | 0% | 0% |

Participants responded also to these questions:

- When did the discomfort begin?
- How long did it last?
- How much did these sensations affect your performance?

All participants that felt a skin sensation reported that the discomfort began at the beginning of the block (except one participant that reported skin sensation at the middle of the block). All participants reported that the discomfort stopped quickly and it did not affect at all the performance.
